# Non-contact seismocardiogram measurement and HRV analysis using cardiac beamforming with FMCW radar

**DOI:** 10.3389/fphys.2025.1733573

**Published:** 2026-01-23

**Authors:** Guang Yu, Chenxi Yang, Haobo Li, Chaochao Wang, Xianchao Zhang, Jianqing Li, Chengyu Liu

**Affiliations:** 1 The State Key Laboratory of Bioelectronics, School of Instrument Science and Engineering, Southeast University, Nanjing, China; 2 The School of Science and Engineering, University of Dundee, Dundee, United Kingdom; 3 The Provincial Key Laboratory of Multimodal Perceiving and Intelligent Systems, Jiaxing University, Jiaxing, Zhejiang, China

**Keywords:** aortic opening (AO) detection, beamforming, frequency-modulated continuous-wave (FMCW) radar, heart rate variability (HRV), interbeat interval (IBI), seismocardiogram (SCG), wavelet packet transform (WPT)

## Abstract

**Introduction:**

Heart rate variability (HRV) is a vital metric for assessing cardiovascular health, psychological stress, and sleep quality. Non-contact HRV monitoring offers advantages in safety, comfort, and hygiene, making it an increasingly attractive solution.

**Methods:**

In this study, we propose a high-precision, non-contact HRV analysis method using a 77 GHz multiple-input multiple-output (MIMO) frequency-modulated continuous wave (FMCW) radar system. The proposed method first employs an optimized Capon beamforming algorithm to accurately localize the heart and enhance intermediate frequency (IF) signals from the heart’s direction. A modified differentiate and cross-multiply (MDACM) algorithm is then used to demodulate the phase sequence, yielding a raw vital sign signal that includes both respiratory and cardiac components. This signal is further processed using a six-level wavelet packet transform (WPT), from which specific wavelet coefficients (6th to 12th bands at level six) are selected to reconstruct the seismocardiogram (SCG) signal. To extract precise inter-beat interval (IBI) sequences, a robust aortic valve opening (AO) point detection algorithm is developed. Time-domain HRV indices—including the standard deviation of normal-to-normal intervals (SDNN), the root mean square of successive differences (RMSSD), and the percentage of successive normal-to-normal intervals differing by more than 50 milliseconds (ms) (pNN50)—are then computed from the IBI sequence. To validate the approach, we developed a synchronized data acquisition system combining radar and electrocardiogram (ECG) sensors and collected data from 13 participants—each person collected data for 10 min.

**Results:**

Experimental results demonstrate the effectiveness of our method, achieving average errors of 4.11 ms in SDNN, 8.05 ms in RMSSD, and 2.15% in pNN50 compared to ECG-derived ground truth.

**Discussion:**

These results outperform existing non-contact HRV monitoring techniques and highlight the method’s potential for practical, continuous, and unobtrusive cardiovascular monitoring.

## 1 Introduction

Heart rate variability (HRV) refers to the variations in successive heartbeat intervals under sinus rhythm. It serves as physiological indicator reflecting the autonomic nervous adaptive capacity to external stimuli and stress. HRV analysis was first introduced by obstetricians Hon and Lee in 1965 (Hon and Lee, 1963), and following successful clinical applications by researchers such as Wolf (Wolf et al., 1978) and Kleiger (Chiou et al., 1997), it gradually became an independent diagnostic criterion for cardiovascular diseases. HRV is now widely utilized in the auxiliary diagnosis of various cardiovascular conditions (Chen, 2002; Pecchia et al., 2011; Li et al., 2019), as well as in the assessment of mental stress (Chalmers et al., 2014) and sleep quality (Penzel et al., 2003; Gil et al., 2009; Hietakoste et al., 2024). HRV analysis methods primarily include time-domain, frequency-domain, and nonlinear methods. Time-domain methods quantitatively analyze the time-domain characteristics of the RR interval series by using traditional statistical approaches to derive HRV indicators. These methods demonstrate strong resistance to interference, making them suitable for long-term detection and analysis, leading to a comprehensive evaluation of the autonomic nervous system’s modulation of heart rate. HRV analysis requires the acquisition of inter-beat intervals (IBI). Traditional HRV analysis typically collects electrocardiogram (ECG) signals to obtain RR interval sequences or utilizes photoplethysmography (PPG) signals to acquire peak-to-peak interval sequences. However, ECG signal collection necessitates attaching electrodes to the skin, which can reduce comfort and lead to skin allergies. Similarly, using wearable devices to collect phonocardiographic (PCG) signals can also pose challenges related to comfort and limited battery life. Accelerometer-based measurements of thoracic vibrations to extract heartbeat signals can also be employed for HRV analysis (Lahdenoja et al., 2016); however, the requirement to place the accelerometer device on the thorax can still result in discomfort for the user. Non-contact measurement of heart signals for HRV analysis offers a comfortable solution that avoids skin contact, thereby eliminating the risk of skin allergies. This approach is particularly advantageous for populations such as burn patients, infants, and those affected by epidemics who may be unsuitable for ECG electrodes or PPG sensors. Consequently, non-contact measurement methods for HRV have garnered significant attention from researchers, resulting in a wealth of study outcomes. The related research primarily encompasses two strategies: remote PPG (rPPG) signal extraction via camera measurements and heartbeat signal extraction using radar technology. Camera-based rPPG signal measurement is vulnerable to variations in lighting conditions and body movements, which makes it challenging to obtain high-quality rPPG signals. This further complicates the precise extraction of IBI from rPPG signals, hindering accurate HRV analysis. Additionally, cameras also raise privacy concerns and are ineffective in low-light or nighttime conditions. In contrast, radar sensor-based approaches employing radio frequency methods for HRV analysis have attracted greater interest.

In the process of measuring vital signs using radar, low-power modulated electromagnetic waves are emitted towards the human body. By analyzing the phase changes between the transmitted signal and the echo, the vibrations of the chest can be detected, allowing for the acquisition of vital signs such as respiration and heartbeat. Building upon the radar-measured heartbeat signals, further analysis of HRV can be conducted, offering advantages such as safety, comfort, and the absence of privacy concerns. The earliest application of radar in detecting vital signs dates back to 1971 (Caro and Bloice, 1971), when Caro at Imperial College London developed a radar-based non-contact respiratory monitoring system for infants to detect apnea and provide early warning. This study marks the first documented application of radar in vital sign detection. Subsequently, interest in radar-based vital sign measurement systems grew among researchers. However, most studies have primarily focused on measuring respiration rate and heart rate (Li et al., 2006; Li et al., 2008; Gu et al., 2010; He et al., 2017; Mercuri et al., 2018; Saeed et al., 2021; Wang et al., 2024; Yang et al., 2024), which lack the capability to extract high signal-to-noise ratio (SNR) heartbeat signals. This limitation hinders the precise determination of heartbeat timings, ultimately affecting the accuracy of HRV analysis. Thus, the accurate measurement of heartbeat signals with precise heartbeat timings using radar sensors is critical for accurate HRV analysis. Although existing research on radar-based heart rate measurement has achieved high accuracy, these studies typically calculate heart rate values in the frequency domain, relying on averaged heart rate over a time window, without capturing complete and precise heartbeat signals. In fact, achieving accurate HRV analysis is considerably more challenging than merely calculating heart rate.

Continuous wave (CW), ultra-wideband (UWB), and frequency modulated continuous wave (FMCW) radar systems are commonly utilized for non-contact measurement of vital signs. In recent years, there has been increasing interest among researchers in the use of FMCW radar operating at millimeter wavelengths for vital sign detection. FMCW millimeter-wave radar modulates continuous millimeter waves to measure chest vibrations based on phase changes. This approach effectively mitigates non-target signal interference by considering the distance and orientation of the subject, and it is capable of detecting vital signs from multiple individuals simultaneously (Wang et al., 2021a). Moreover, FMCW millimeter-wave radar features a compact size, high integration, and low transmit power, making it particularly suitable for vital sign monitoring. The heartbeats and respiratory movements induce subtle vibrations in the chest, which the radar detects by demodulating the phase changes in I/Q signals. However, challenges remain in employing radar for heart signal measurement and HRV analysis:

1. The heartbeat signal is strongest at the anatomical location of the heart. Accurately localizing the heart position using radar can effectively suppress echo interference from other directions and enhance the echo signal originating from the heart, thereby enabling the acquisition of heartbeat signals with the highest SNR. However, although existing studies utilizing beamforming techniques can detect the presence and general location of the human body, they face challenges in precisely localizing the heart and extracting high-SNR heartbeat signals.
2. The heartbeat-induced chest displacement is extremely subtle compared to that caused by respiration. Specifically, respiration typically induces a displacement of approximately 3–12 mm, whereas the displacement due to cardiac activity is only about 0.1–0.5 mm. As a result, the heartbeat signal is easily masked by the respiratory signal and ambient noise. This makes the separation of heartbeat signals from raw vital sign data highly challenging and consequently impedes accurate HRV analysis. Developing algorithms capable of effectively separating heartbeat and respiratory components remains an ongoing research challenge.

To address the challenges associated with radar-based heart rate variability (HRV) analysis, this study proposes a novel HRV analysis framework based on a 77-GHz FMCW radar system. The proposed method employs an optimized beamforming technique to accurately localize the cardiac region, effectively suppressing echo signals from non-cardiac directions while enhancing those originating from the heart. As a result, high signal-to-noise ratio (SNR) remote seismocardiography (SCG) signals can be extracted. Based on the recovered SCG signals, the aortic valve opening (AO) points are detected, from which the inter-beat interval (IBI) sequence is derived for subsequent HRV analysis. The HRV metrics include the root mean square of successive differences (RMSSD), the standard deviation of normal-to-normal intervals (SDNN), and the percentage of successive normal interval differences exceeding 50 ms (pNN50). Experiments were conducted with synchronous radar and ECG acquisitions, and the proposed method was compared with four state-of-the-art heartbeat extraction approaches: a Butterworth bandpass filter (BPF), which has been widely adopted in numerous studies (Petrović et al., 2019; Xia et al., 2018), the mmHRV method (Wang et al., 2021b), the variational mode decomposition (VMD) method (Yu et al., 2023), and the cyclostationary singular spectrum analysis (CiSSA) method (Hu et al., 2021). The absolute errors of the HRV indices obtained by these five methods with respect to the ECG-derived HRV metrics were calculated as quantitative accuracy measures. In addition, the cardiac localization performances of three classical beamforming methods and the proposed optimized Capon beamforming method were visually compared. The experimental results demonstrate that the proposed optimized Capon beamforming technique can accurately localize the cardiac direction, and that the radar-based HRV analysis results obtained using the proposed framework show the closest agreement with those derived from the synchronous ECG signals. The main contributions of this work are summarized as follows:

1. We propose an optimized Capon beamforming approach for localizing the heart position, in which a signal quality index (SQI) for the SCG is introduced to replace the conventional azimuth spectrum function used in Capon beamforming. This index is employed to search for the azimuth angle corresponding to the accurate heart location.
2. We applied the wavelet packet transform (WPT) (Coifman and Wickerhauser, 1992) algorithm to extract the SCG signal containing accurate heartbeat information from the vital signs.
3. We designed a robust AO detection algorithm that effectively prevents the misidentification of other peaks as AO points, facilitating precise measurement of the IBI.
4. We compared four of the most popular heartbeat signal extraction methods and evaluated their performance differences.

The remaining sections of this paper are organized as follows: Chapter 2 introduces related research work, Chapter 3 provides a detailed description of the proposed methods, Chapter 4 presents the experimental procedures and results, Chapter 5 offers a discussion, and the final chapter concludes the study.

## 2 Related work

The non-contact measurement of heart rate and respiration based on radar has been extensively studied, achieving high accuracy in heart rate estimation. However, research on radar-based HRV analysis is relatively limited. HRV analysis provides a wealth of physiological indicators beyond heart rate, which are crucial for understanding human health. Numerous studies have attempted to utilize radar technology for non-contact HRV analysis. In 2009, Massagram et al. (2009) and colleagues employed a direct conversion quadrature radar system combined with linear demodulation techniques to achieve high-precision HRV analysis and the extraction of the respiratory sinus arrhythmia index (RSA). Experimental results indicated that the standard deviation differences in normal inter-beat interval indices derived from Doppler radar and ECG references were all less than 9 ms. In 2014, Hu et al. (2014) utilized continuous wavelet filtering and ensemble empirical mode decomposition (EEMD) algorithms to recover and separate the original cardiopulmonary signals obtained by radar, thereby facilitating precise beat interval extraction in the time domain for HRV analysis. The results demonstrated that the relative error in the extracted heartbeat intervals, compared to ECG R-R peak intervals, ranged from 2.53% to 4.83%. In 2019, Petrović et al. (2019) introduced a novel algorithm to estimate HRV features using a 24 GHz continuous wave Doppler radar structured in quadrature, processing the combined I/Q signals through a filter bank of narrowband bandpass filters with varying center frequencies. Based on rough heart rate estimates, one bandpass filter output was selected as the effective output. The zero crossings of the filtered output signals represented heartbeats and were used to extract IBI. Ultimately, four HRV characteristics were calculated from the IBIs, with the algorithm tested on real recorded data from ten subjects. The average relative error in the extracted IBIs compared to ECG measurements was between 1.02% and 2.07%. In 2020, Antolinos et al. (2020) employed a 122 GHz FMCW radar to monitor vital signs, utilizing the empirical mode decomposition (EMD) algorithm to separate heartbeat and respiration signals for HRV analysis based on the heartbeat signal. That same year, Wang et al. (2021b) proposed a millimeter-wave radar HRV analysis system (mmHRV), which included a heartbeat signal extractor designed to optimize the phase decomposition of chest motion modulation channel information. This enabled the accurate estimation of heartbeat signal timing by identifying peak positions, allowing further derivation of IBIs for HRV assessment. Experimental results indicated that mmHRV accurately measured HRV, with a median IBI estimation error of 28 ms (96.16% accuracy). In 2021, Xia et al. (2021) introduced a decoding peak detection (DPD) method to address the challenge of extracting heartbeat peaks by decoding the most probable state sequences from the single-band frequency envelope (FEnv) of radar signals, thereby facilitating HRV analysis. Additionally, a hidden semi-Markov model (HSMM) was utilized to detect heartbeat timings within bandpass-filtered radar data. Evaluation against ECG as the gold standard demonstrated an average F1 score of 93.19% ± 0.73% across six subjects. The accuracy of the IBIs extracted from radar was assessed with average relative errors of 0.51%–1.06% for HSMM and 0.37%–1.15% for DPD. In 2023, Yu et al. (2023) proposed a novel radar-based heartbeat detection method that extracts high-fidelity pulse templates from smoothed PCG waveforms through template matching to analyze HRV based on radar-derived heart sound signals.

Existing radar-based HRV analysis methods often face challenges in accurately localizing the heart and extracting high SNR non-contact SCG signals, leading to substantial errors in heartbeat localization. Achieving high-precision HRV analysis with radar critically depends on accurate localization of the heart, reliable measurement of high-SNR cardiac signals, and precise detection of individual heartbeats. In this work, we propose an HRV analysis method based on a 77 GHz FMCW radar system that not only enables precise beam steering toward the heart but also effectively separates high-SNR non-contact SCG signals from raw vital sign data. Furthermore, we develop a robust AO point detection algorithm capable of accurately identifying the AO fiducial points, thereby enabling the extraction of highly precise IBI sequences. The proposed method achieves high accuracy in time-domain HRV metrics.

## 3 Methods

**FIGURE 1:**
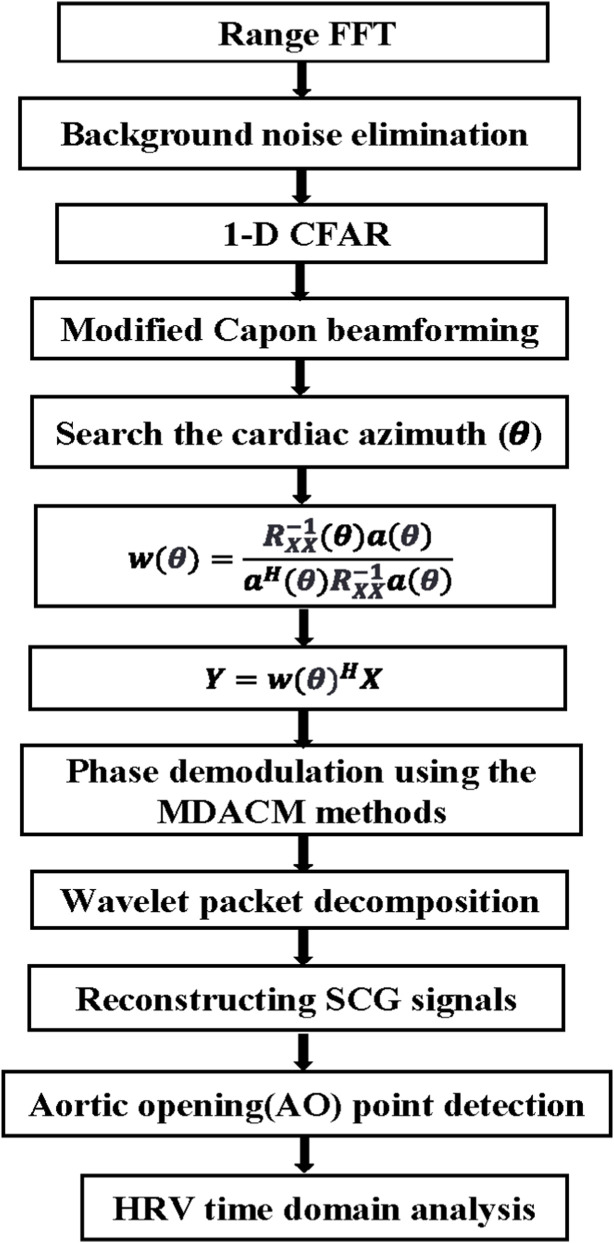
Flowchart of the proposed method.

High-precision radar-based HRV analysis relies heavily on heartbeat signals with a high SNR. The proposed method for remote SCG (rSCG) signal extraction is illustrated in the flowchart shown in Figure 1. A commercially available Texas Instruments AWR1642 radar sensor is employed and configured in a 2-transmit 4-receive (2T4R) mode via time-division multiplexing (TDM). The rSCG signal is extracted from the raw intermediate frequency (IF) data sampled by the radar’s analog-to-digital converter (ADC).

First, a fast-time Fourier transform (FFT) is performed on the IF signals to obtain range-bin data for all eight antennas. To suppress static background interference, the mean value of each range bin is subtracted from itself. Then, for each column vector along the fast-time dimension of the range-bin matrix, the index corresponding to the maximum energy is identified, and the complex value at that index is extracted to form a slow-time complex sequence at the range gate corresponding to the human target. Next, an optimized Capon beamforming algorithm is employed to search for the optimal direction of the heartbeat signal, from which the optimal spatial weight vector is derived. This weight vector is then used to perform a weighted summation of the slow-time complex sequences across the eight antennas at the selected range gate. Finally, a phase demodulation technique termed modified differential arctangent cross-multiplication (MDACM) (Xu et al., 2021) is applied to the resulting complex sequence to extract its phase, which constitutes the vital sign signal containing both respiration and heartbeat components.

Subsequently, a six-level wavelet packet decomposition (Coifman and Wickerhauser, 1992) is applied to the vital sign signal, and the wavelet coefficients within the frequency band corresponding to SCG are selected and reconstructed to obtain the rSCG signal. We further design a simple and efficient aortic valve opening (AO) peak detection algorithm with high localization accuracy. Once the continuous AO point sequence is obtained, IBIs can be readily derived, enabling the calculation of time-domain HRV metrics.

### 3.1 FMCW millimeter wave radar signal model

The relationship between the frequency of the Chirp signal emitted by the FMCW millimeter-wave radar and time is expressed by Equation 1:

$$f\left(t\right)=f_{c}+\frac{B}{T_{c}}t$$ (1)

Where $f_{c}$ is the frequency at the initial moment when the chirp signal is transmitted, $T_{c}$ is the duration of the chirp signal frequency sweep, and B is the frequency modulation bandwidth. $\emptyset_{1}$ is initial phase. Therefore, the FMCW millimeter wave radar transmission signal can be expressed as Equation 2:

$$x_{T}\left(t\right)=A_{T}\cos\left(2\pi f_{c}t+\pi \frac{B}{T_{c}}t^{2}+\emptyset_{1}\right)$$ (2)

Assuming the distance between the radar and the target person is R , the radar emits a chirp signal that reaches the chest of the person and then reflects back. The round trip time is expressed by Equation 3:

$$t_{d}=\frac{2R}{c}$$ (3)

Where c is the speed of light, then the expression of the reflected echo signal is expressed by Equation 4:

$$x_{R}\left(t\right)=x_{T}\left(t-t_{d}\right)=A_{R}\cos\left(2\pi f_{c}\left(t-t_{d}\right)+\pi \frac{B}{T_{c}}{\left(t-t_{d}\right)}^{2}+\emptyset_{2}\right)$$ (4)

The internal circuit of the radar mixes the transmitted signal and the reflected echo signal to obtain an IF signal in expression (Equation 5):

$$x_{IF}\left(t\right)=x_{T}\left(t\right)\cdot x_{R}\left(t\right)$$ (5)

From the product and difference formula of trigonometric functions, we can get:

$$x_{IF}\left(t\right)=\frac{A_{T}\cdot A_{R}}{2}\cos\left(\frac{4\pi BR}{cT_{c}}t+\frac{4\pi f_{c}R}{c}+\frac{4\pi BR^{2}}{c^{2}T_{c}}t^{2}+\emptyset \right),\emptyset =\emptyset_{1}-\emptyset_{2}$$ (6)

Since $4\pi BR^{2}$ is much smaller than $c^{2}T_{c}$ , and the chirp signal period is very short, $t^{2}$ is also close to zero. Therefore, the quadratic term $\frac{4\pi BR^{2}}{c^{2}T_{c}}t^{2}$ in expression (Equation 6) is almost equal to zero and can be ignored. At the same time, let $A_{IF}=A_{T}\cdot A_{R}/2$ , then the simplified IF signal expression is Equations 7, 8:

$$x_{IF}\left(t\right)=A_{IF}\cos\left(\frac{4\pi BR}{cT_{c}}t+\frac{4\pi f_{c}R}{c}+\emptyset \right)$$ (7)

$$x_{IF}\left(t\right)=A_{IF}\cos\left(f_{IF}t+\varphi_{IF}+\emptyset \right)$$ (8)

$$f_{IF}=\frac{2B}{c\cdot T_{c}}\cdot R$$ (9)

$$\varphi_{IF}=\frac{4\pi f_{c}R}{c}=\frac{4\pi }{\lambda }\cdot R$$ (10)

Where $f_{IF}$ is the frequency of the IF signal in Equation 9. $\varphi_{IF}$ is the phase of the IF signal. λ is the wavelength of the electromagnetic wave emitted by the millimeter wave radar. After the phase expression (Equation 9) is differentiated, the phase change of the IF signal with respect (Equation 10) to the distance is expressed as:

$$\Delta \varphi_{IF}=\frac{4\pi }{\lambda }\cdot \Delta R$$ (11)

Where $\Delta \varphi_{IF}$ is the phase change of the IF signal, and ΔR is the change in chest vibration displacement. From Equation 11, it is evident that when the initial frequency of the millimeter-wave radar is 77 GHz, the wavelength λ≈4 mm, resulting in a phase shift of 0.314 radians for a chest displacement of 0.1 mm induced by the heartbeat. Thus, we can capture the minute chest vibrations caused by heartbeat and respiration through significant phase variations. This principle forms the foundation for using FMCW millimeter-wave radar to measure human vital sign signals.

### 3.2 Cardiac azimuth search and vital signs extraction

When the radar transmits chirp signals toward the human body from a certain angle, the reflected waves from the thoracic cavity may scatter in multiple directions, and only a portion of these echoes are received by the radar antennas. Beamforming can effectively enhance the echo signals from the desired direction while suppressing clutter and interference from other directions. Beamforming relies on a multiple-input multiple-output (MIMO) antenna array. The AWR1642 mm-wave radar used in this study can be configured as a 2-transmit, 4-receive (2T4R) antenna array, which is virtually extended to a 1-transmit, 8-receive (1T8R) linear array using TDM. To enhance the echo signal from the thoracic cavity and reduce the output noise and interference power, we adopt the Capon beamforming method (Capon, 1969). Capon beamforming, also known as minimum variance distortionless response (MVDR) beamforming, can effectively suppress clutter signals from non-target directions while enhancing signals from the desired direction. It provides superior angular resolution performance compared to conventional beamforming methods (Marino and Chau, 2005), and although its resolution is lower than that of the multiple signal classification (MUSIC) algorithm (Schmidt, 1986), Capon beamforming offers significantly lower computational complexity. In the 1T8R uniformly spaced linear antenna array, the IF signal received at each antenna element experiences a time delay relative to the adjacent previous antenna. Let the time delay be denoted as τ , d be the inter-element spacing, and θ be the angle of arrival (AoA) of the incoming signal, then τ is expressed by Equation 12:

$$\tau =\frac{dsin\left(\theta \right)}{c}$$ (12)

The slow-time complex sequence corresponding to the range bin of the human target can be reasonably modeled as a narrowband signal. Due to the different propagation paths between the target and individual receiving antennas, the signal phase exhibits variations across antenna elements within the inter-antenna propagation delay range. Let the IF signal at the target range bin have a frequency of $f_{h}$ , an amplitude of $A_{h}$ , and a phase of $\varphi_{h}$ . At a given time instant t , assuming the presence of zero-mean Gaussian noise $n_{m}\left(t\right)$ , and denoting the signal amplitude received by the m-th antenna as $A_{h}\left(m\right)$ , the signal received by the m-th antenna can be expressed as Equation 13:

$$\begin{array}{ll} x_{m}\left(t\right) & =A_{h}\left(m\right)\cdot \cos\left(2\pi f_{h}\left(t-m\tau \right)+\varphi_{h}\right)+n_{m}\left(t\right)\\ & =A_{h}\left(m\right)\cdot \cos\left(2\pi f_{h}t+\varphi_{h}\right)\cdot \cos\left(-2\pi f_{h}\cdot m\tau \right)+n_{m}\left(t\right)\;m\\ & =0,1,2,3,4,5,6,7. \end{array}$$ (13)

Let $s\left(t\right)=A_{h}\left(m\right)\cdot \cos\left(2\pi f_{h}t+\varphi_{h}\right)$ , $a_{m}\left(\theta \right)=\cos\left(-2\pi f_{h}\cdot m\frac{dsin\left(\theta \right)}{c}\right)$

$$\begin{array}{ll} a\left(\theta \right) & ={\left[a_{0}\left(\theta \right),a_{1}\left(\theta \right),a_{2}\left(\theta \right),\ldots \ldots \ldots ..,a_{7}\left(\theta \right)\right]}^{T},\\ & \;n\left(t\right)={\left[n_{0}\left(t\right),n_{1}\left(t\right),n_{2}\left(t\right),\ldots \ldots \ldots ..,n_{7}\left(t\right)\right]}^{T} \end{array}$$

The target range gate signals (as snapshot signals) from the eight virtual antennas are arranged into a vector form as Equation 14:

$$X\left(i\right)=s\left(i\right)a\left(\theta \right)+n\left(i\right),i=0,1,2,3,\ldots ..N$$ (14)

Where *N* is the number of snapshots. The calculation formula of Capon beamforming weight vector is as Equation 15:

$$w\left(\theta \right)=\frac{R_{XX}^{-1}\left(\theta \right)a\left(\theta \right)}{a^{H}\left(\theta \right)R_{XX}^{-1}a\left(\theta \right)}$$ (15)

The spatial spectrum function is expressed by Equation 16:

$$P\left(\theta \right)=\frac{1}{a^{H}\left(\theta \right)R_{XX}^{-1}a\left(\theta \right)}$$ (16)

$w\left(\theta \right)$ is the optimal weight vector, $R_{XX}$ is the snapshot signals covariance matrix, and $R_{XX}^{-1}$ represents the inverse matrix of the snapshot signal covariance matrix. There is expressed by Equation 17:

$$R_{XX}=\frac{1}{N}\sum_{i=1}^{N}\;\left[X\left(i\right)X^{H}\left(i\right)\right]$$ (17)

The weighted sum of the target range gate slow-time complex signals of the eight virtual antennas is performed, and the expression of the enhanced slow-time complex signal is obtained as Equation 18:

$$Y=w^{H}X$$ (18)

As shown in Formula 18, the slow-time complex sequence signals of the target range gates from all eight antenna elements can be weighted summed to enhance the signal from the direction of the human body while suppressing clutter signals from other directions. However, the direction corresponding to the peak of the spatial spectrum function in Equation 16 does not necessarily coincide with the direction of the highest SNR of the heartbeat signal. In some cases, the spectral peak may occur in the direction of clutter or other objects. Furthermore, even if the spectral peak appears in the direction of the human body, it does not guarantee that this is the direction with the highest SNR for the heartbeat signal. For most individuals, whose body height range is about 1.55–1.9 m, only the region around the heart yields heartbeat signals with the highest SNR. Additionally, in some individuals who exhibit abdominal breathing, significant motion occurs in the abdominal region. Conventional beamforming methods combined with respiration detection (Wang et al., 2021b) may misidentify the abdominal region as the target direction, which still does not correspond to the direction of the strongest heartbeat signal. To address these limitations, we propose a new azimuth search method to replace the traditional spatial spectrum function approach, aiming to identify the direction with the highest SNR of the SCG signal. At the beginning of the measurement, we collect the first 20 s of data and process them using the procedure outlined in Figure 1 to extract the SCG signal. To assess the quality of SCG signals, we propose a method based on dynamic time warping (DTW) (Sakoe and Chiba, 1978). Specifically, a 20 s segment with high-quality SCG signal, selected from all radar-measured SCG recordings, is designated as the template signal. The DTW distance is then calculated between the SCG signal under evaluation and the template signal. The beamforming angle is selected by minimizing the DTW distance between the extracted SCG signal and the reference template. A smaller DTW distance indicates a higher similarity between the two signals, thereby reflecting better signal quality. Conversely, a larger DTW distance suggests poorer signal quality. Let the selected template signal be denoted as ${SCG}_{template}$ , and the SCG signal to be evaluated as SCG . Prior to DTW computation, both SCG and ${SCG}_{template}$ are standardized. Here, σ represents calculating the variance of the SCG sequence as shown in (Equations 19, 20):

$$SCG=\frac{SCG-\bar{SCG}}{\sigma \left(SCG\right)}$$ (19)

$${SCG}_{template}=\frac{{SCG}_{template}-\bar{{SCG}_{template}}}{\sigma \left({SCG}_{template}\right)}$$ (20)

$$SQI=\frac{1}{DTW\left({SCG,SCG}_{template}\right)}$$ (21)

To identify the direction with the highest SNR of the SCG signal, we perform a directional search over the angular range of [-60°,60°] with a step size of 1°. For each angle, a corresponding beamforming weight vector w is computed, and based on this, a SQI value is calculated, as shown in Equation 21. This process yields a total of 121 angle-SQI pairs. Among all scanned directions, the angle $\theta_{opt}$ corresponding to the maximum SQI value is selected, which we consider to be the direction with the highest SCG signal SNR. Upon determining the cardiac orientation, the SCG template is no longer utilized, and the search for the optimal orientation via improved Capon beamforming is terminated. The resulting beamforming azimuth is subsequently fixed for all further processing. After determining $\theta_{opt}$ , the optimal beamforming weight vector $w_{opt}$ is calculated according to Formula 15, and the slow-time complex signal in the direction of the heart is obtained using Formula 18. This slow time series is then demodulated using a method called MDACM (Xu et al., 2021) to produce a phase series containing respiratory and cardiac motion components, i.e., the original vital sign signal.

### 3.3 SCG signal extraction

**FIGURE 2:**
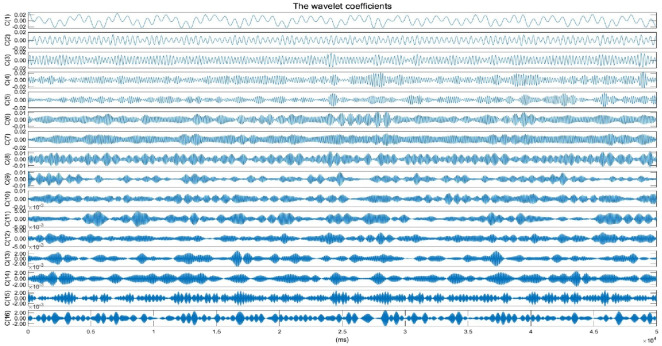
The wavelet coefficients (only the first 16 wavelet coefficients are plotted).

The original vital sign signal, primarily contains the heartbeat signal, respiratory signal, and other noise. Due to the greater displacement of the respiratory signal compared to that of the heartbeat signal, the heartbeat signal is often overshadowed by the respiratory signal. Additionally, the third harmonic frequency band of the respiratory signal overlaps with the frequency band of the heartbeat signal, complicating the separation of these two signals. Designing an algorithm to effectively separate the heartbeat signal from the respiratory signal remains a challenge. Commonly used methods, such as the BPF, typically set parameters of 0.1 Hz–0.5 Hz for the respiratory signal and 0.7 Hz–3 Hz for the heartbeat signal. However, these methods yield only moderate results and fail to address the issue of frequency band overlap caused by the respiratory signal’s third harmonic. Standard binary wavelet analysis decomposes signals layer by layer along the low-frequency direction, which limits its ability to finely segment the frequency bands, particularly in the high-frequency region. The heartbeat signal contains both high-frequency and low-frequency components, and binary wavelet analysis is insufficient for capturing the entire frequency spectrum of the heartbeat signal. Wavelet packet analysis offers a more detailed and nuanced approach to signal analysis. Unlike standard binary wavelet analysis, which analyzes low and high-frequency components separately, wavelet packet decomposition simultaneously considers both, providing a more accurate local analysis. This method allows for finer division of the time-frequency plane and offers superior resolution for the high-frequency components of the signal. Moreover, wavelet packet analysis introduces the concept of optimal basis selection, allowing for adaptive selection of the most suitable basis functions that align with the characteristics of the signal, thereby significantly enhancing the effectiveness of the signal analysis. In this study, we employ WPT using the “db45” wavelet to break down the original vital sign signal into a series of finely segmented wavelet packet coefficients within specific frequency ranges. The “db45″wavelet has a 45th-order vanishing moment. A higher vanishing moment means a higher concentration in the frequency domain, which can better capture the high-frequency details of the signal by selecting the wavelet packet coefficients corresponding to the SCG signal frequency range, we reconstruct the SCG signal. The original vital sign signal, sampled at a rate of $f_{s}=200$ Hz over a duration of 50 s, undergoes six levels of decomposition using the WPT. The first 16 wavelet coefficients from the sixth level are illustrated in Figure 2. According to the WPT theory, the frequency range of the i-th wavelet coefficient $C\left(i\right)$ of the sixth layer is expressed by Equation 22

$$C\left(i\right)∼\left[\left(i-1\right)\cdot \frac{f_{s}/2}{2^{6}},i\cdot \frac{f_{s}/2}{2^{6}}\right]$$ (22)

**FIGURE 3:**
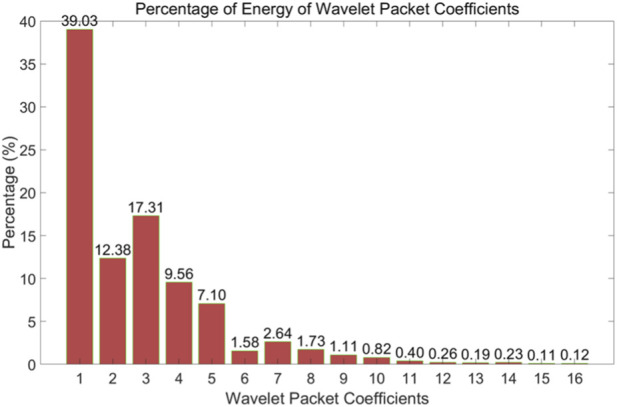
Energy percentage histogram of wavelet packet coefficients.

**FIGURE 4:**
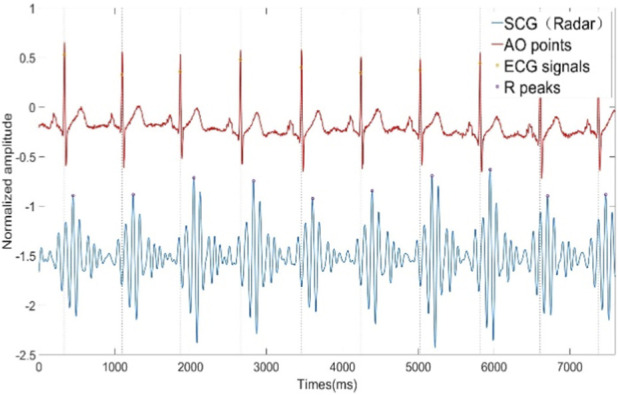
SCG signal extracted by WPT and ECG signal collected synchronously.

**FIGURE 5:**
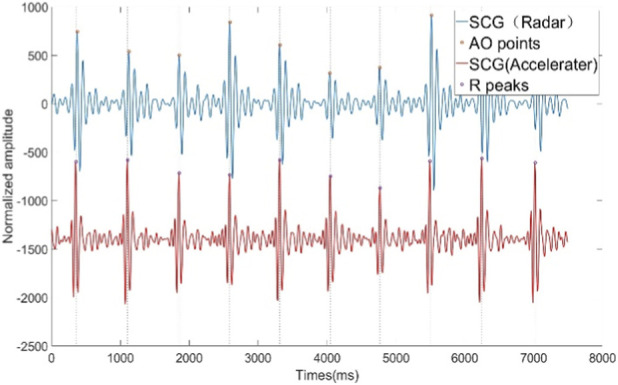
SCG signals extracted from radar signals and SCG signals from synchronized acceleration measurements.

Where $f_{s}=200$ Hz is the sampling rate of the original vital signs signal. Further analysis of the wavelet packet coefficient energy percentage was conducted, with results illustrated in Figure 3. The energy distribution shows that the first five wavelet packet coefficients account for a significantly higher energy percentage compared to the remaining coefficients. According to Equation 22, the frequency range for the first wavelet packet coefficient spans from 0 Hz to 1.5625 Hz, primarily containing respiratory components and motion artifacts. The second through fourth wavelet packet coefficients mainly consist of harmonic components of respiratory and motion artifacts. From the sixth wavelet packet coefficient to approximately the 12th, a peak in energy appears, indicating that these six coefficients primarily capture the heartbeat signal components. Although the first five wavelet packet coefficients may contain some heartbeat signal components, the heartbeat signal content in these coefficients is significantly lower than that of respiratory components, motion artifacts, and their harmonics. Literature (Taebi et al., 2019) reports that the primary frequency range of the SCG signal is below 25 Hz. Additionally, literature Equation 22, the frequency range for these coefficients is 7.8125 Hz–18.750 Hz, which closely aligns with the SCG frequency range described in (Zhang et al., 2023). Therefore, the selected wavelet packet coefficients effectively reconstruct the SCG signal. The time-domain waveform and the synchronized ECG are shown in Figure 4. Figure 5 shows the SCG signal extracted from the radar signal and the SCG signal measured from the synchronously acquired cardiac acceleration signal. As shown in Figure 5, the SCG signal measured by radar exhibits a high degree of similarity to the SCG signal measured by an accelerometer attached near the skin overlying the heart.

### 3.4 AO point detection of SCG signal

**FIGURE 6:**
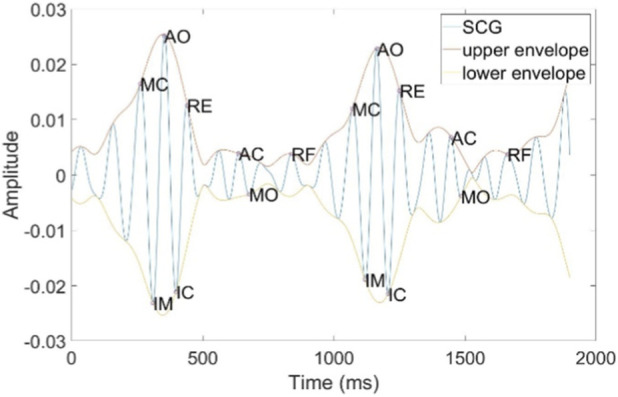
SCG waveform and important key points.

The SCG is generated by micro-vibrations of the thoracic cavity caused by cardiac contractions. It consists of several peaks and troughs that encompass physiological information related to the heart, including the following features: Atrial Systole (AS), Mitral Closure (MC), Isovolumetric Contraction (IM), Aortic Opening (AO), Isovolumetric Relaxation (IC), Rapid Ejection (RE), Aortic Closure (AC), Mitral Opening (MO), and Rapid Filling (RF), as illustrated in Figure 6. Among these, the AO point is characterized by its distinct features, making it easier to detect. We have designed a method for accurately locating the AO point, which is both simple and efficient. The detailed steps of the algorithm are outlined in Algorithm 1. The algorithm was developed in the MATLAB 2021b environment. Initially, the Hilbert Transform (HT) is employed to calculate the upper and lower envelope signals of the SCG. The MATLAB function findpeaks is then utilized to detect the peaks of the upper envelope and the troughs of lower envelope, with a minimum spacing parameter set to $\text{delta}=0.6*f_{s}$ . To address potential noise factors that may lead to false cardiac cycle segments in the SCG signal, we apply a criterion based on the absolute difference between the x-coordinates of the peaks of the upper envelope and the troughs of the lower envelope. If this absolute difference exceeds $\text{delta}1=0.15*f_{s}$ , we discard the detection of the AO point within that cardiac cycle segment. We observed that the IM and IC points are distinctly characterized as the lowest and second-lowest points within each valid cardiac cycle segment. For each qualified trough of the lower envelope, we first identify the lowest and second-lowest points, which correspond to the IM and IC points, respectively. The AO point is always located within the interval between the IM and IC points. Therefore, we continue to search for the maximum value within the x-coordinate range defined by the IM and IC points, which represents the AO point. This method effectively prevents the erroneous detection of the MC and RE points as AO points, as well as mitigating the risk of falsely identifying peaks from noisy cardiac cycle segments as AO points.

Algorithm 1 **AO Detection Algorithmic Framework.**

$\mathrm{Input}:f_{s},\text{SCG}\left[k\right],k=1,2,3,..L,L=\text{length}\left(\text{SCG}\right)$

**Set** $\text{delta}1=0.15*f_{s}$ , $\text{delta}2=0.1*f_{s}$ .
Calculate the upper and lower envelope signals of $\text{SCG}\left[k\right]$ by Hilbert Transform.

$\text{yupper}\left[k\right],\text{ylower}\left[k\right]=\text{Envelope}\left(\text{SCG}\left[k\right]\right)$

Finding the peak values of the $\text{yupper}\left[k\right]$ and the valley values of $\text{ylower}\left[k\right]$

$\left[y1,x1\right]=Findpeaks\left(\text{yupper}\left[k\right]\right)$

$\left[y2,x2\right]=Findpeaks\left(-\text{ylower}\left[k\right]\right)$

$y2=-y2,N=length\left(x1\right)$

for i=1:N do

$if\;\left|x1\left(i\right)-x2\left(i\right)\right|<\text{delta}1\text{ then}$

$a=x2\left(i\right)-\text{delta}2$

b $=x2\left(i\right)+\text{delta}2$

${SCG}_{temp}=SCG\left[a:b\right]$

$x_{\min1}=\mathrm{argmin}\left({SCG}_{temp}\right)$

$x21\left(i\right)=a+x_{\min1}$

${SCG}_{temp}\left[x_{\min1}\right]=\max\left({SCG}_{temp}\right)$

$x_{\min2}=\mathrm{argmin}\left({SCG}_{temp}\right)$

$x22\left(i\right)=a+x_{\min2}$

$if\;x21\left(i\right)<x22\left(i\right)\text{ then}$

${SCG}_{temp}=SCG\left[x21\left(i\right):x22\left(i\right)\right]$

else

${SCG}_{temp}=SCG\left[x22\left(i\right):x21\left(i\right)\right]$

end if

$x_{\max}=\mathrm{argmax}\left({SCG}_{temp}\right)$

$x_{AO}\left(i\right)$ = $\min\left\{x21\left(i\right),x22\left(i\right)\right\}+x_{\max}$

end if

end for

$\text{Output}:x_{AO}$

### 3.5 HRV time-domain analysis

HRV analysis metrics can be derived from the IBI sequence. In this study, we utilize the three most commonly used time-domain metrics for HRV analysis: SDNN, RMSSD, and pNN50. SDNN represents the standard deviation of all normal sinus beats’ IBI. A higher SDNN indicates greater HRV, reflecting overall autonomic nervous system activity. RMSSD is the square root of the mean squared differences of successive IBI and is primarily used to reflect vagal (parasympathetic) activity; higher RMSSD values indicate increased vagal activity. pNN50 denotes the percentage of successive IBI differences greater than 50 ms, which, similar to RMSSD, reflects vagal activity. A higher pNN50 percentage suggests a stronger regulatory influence from the vagal nerve. The time-domain statistical expressions for SDNN, RMSSD, and pNN50 in HRV analysis are as Equations 23–25:

$$SDNN=\sqrt{\frac{1}{N}\sum_{i=1}^{N}\;{\left(IBI\left(i\right)-\bar{IBI}\right)}^{2}}$$ (23)

$$RMSSD=\sqrt{\frac{1}{N-1}\sum_{i=2}^{N}\;{\left(IBI\left(i\right)-IBI\left(i-1\right)\right)}^{2}}$$ (24)

$$\text{pNN}50=\frac{\sum_{i=2}^{N}\;\left\{\left(\text{IBI}\left(i\right)-\text{IBI}\left(i-1\right)\right)>50\text{ms}\right\}}{N}$$ (25)

Where $\bar{\text{IBI}}$ represents the mean of the IBI sequence, and N represents the length of the IBI sequence.

## 4 Experiments and results

### 4.1 Experiments

**FIGURE 7:**
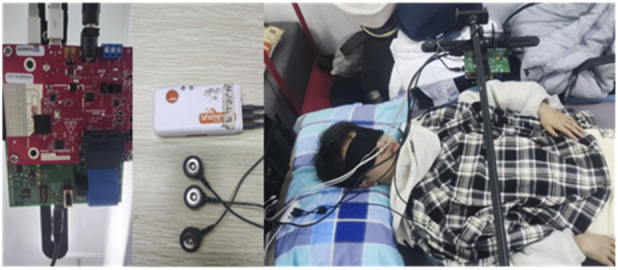
Radar sensor, Shimmer ECG sensor and experimental scene.

We utilized the Texas Instruments AWR1642Boost commercial millimeter-wave radar development kit and the DCA1000EVM data acquisition board to collect reflected echo data from the human thorax. The raw IF data from the radar was transmitted to a laptop via Ethernet. Additionally, we employed the Shimmer ECG sensor to collect ECG signals synchronously as a gold standard reference. A synchronization data acquisition program for the AWR1642 mm-wave radar sensor and Shimmer ECG sensor was developed using MATLAB R2021b. AWR1642 millimeter wave radar antenna uses TDM method to set up two transmit four receive mode, with the Chirp parameters specified in Table 1. A total of 13 participants were involved in the experiment, each person collected data for 10 min. The ages of the participants ranged from 22 to 34 years, and they were dressed in regular t-shirts while lying on a bed. The radar sensor was positioned 0.6 m above the participants, with ECG electrodes attached. Upon starting the MATLAB program and setting the acquisition time, data could be collected for the predetermined duration. The configurations of the radar sensor, Shimmer ECG sensor, and the experimental setup are illustrated in Figure 7. All data processing and algorithm development were conducted within the MATLAB R2021b environment. The primary computer hardware included an Intel i5-10400 CPU, 16 GB of RAM, a 500 GB solid-state drive, and a 1 TB mechanical hard drive.

**TABLE 1:** Chirp parameters of AWR1642 radar.

Parameters	Values	Parameters	Values
Start Frequency	77 GHz	TxStartTime	1 us
IdleTime	8 us	AdcSampleRate	5,000 ksps
AdcStartTime	6 us	RX Gain	30 dB
RampEndTime	60 us	FramePeriodicity	5 ms
FrequencySlope	60MHz/us	Number of Chirp Loops	1

### 4.2 Results

#### 4.2.1 Azimuth estimation for cardiac beamforming

**FIGURE 8:**
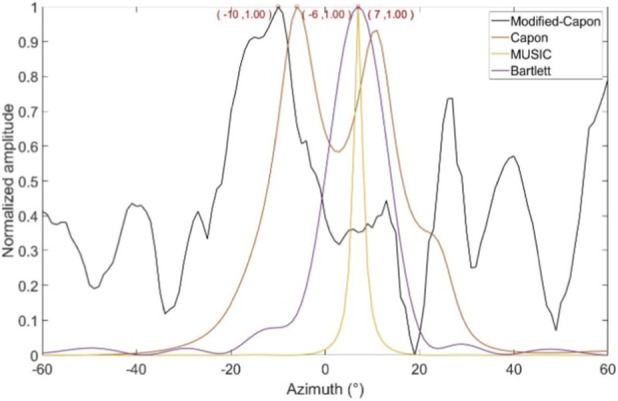
Azimuth angles searched by different beamforming methods (Radar is above the chest).

**FIGURE 9:**
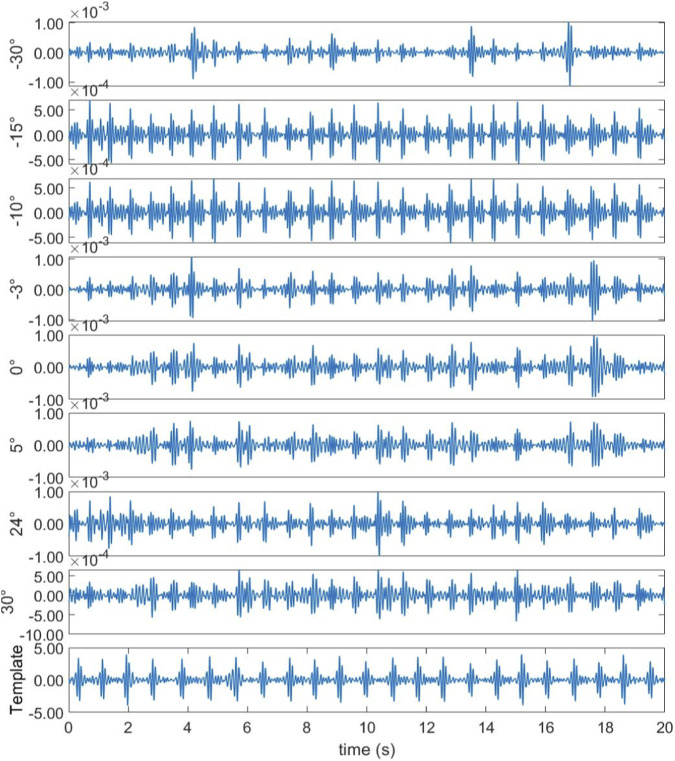
Template signal and SCG signal waveform measured at different azimuths (Radar is above the chest).

In the first experimental scenario, the radar was positioned 0.6 m above the subject’s chest. Figure 8 illustrates the direction-of-arrival (DoA) estimation results obtained using the proposed optimized Capon beamforming algorithm, compared with three classical beamforming methods: MUSIC, Capon, and Bartlett. Figure 9 presents the 20 s time-domain waveforms of SCG signals measured along the estimated directions using each method. The proposed Modified-Capon method estimated the heart direction at −10°, which yielded the highest signal quality SCG waveform among all tested directions. In contrast, MUSIC and Bartlett both estimated the heart direction at 7°, while the Capon method estimated it at −6°. As shown in Figure 8, SCG signals obtained from 7° and −6° exhibit significantly lower quality compared to those measured at −10°.

**FIGURE 10:**
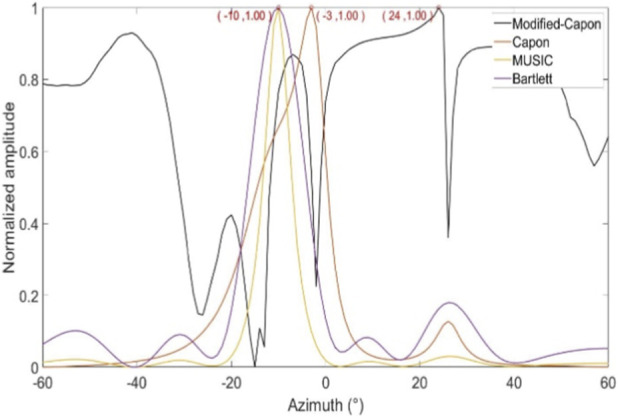
Azimuth angles searched by different beamforming methods (Radar is above the head).

**FIGURE 11:**
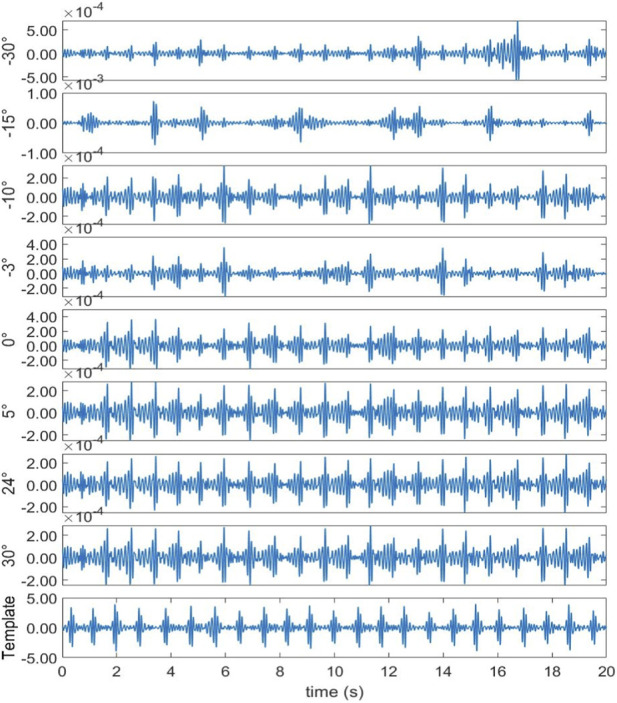
Template signal and SCG signal waveform measured at different azimuths (Radar is above the head).

In the second experimental scenario, the radar was placed 0.6 m above the subject’s head. Figure 10 shows the DoA estimation results for the same four beamforming methods. Figure 11 illustrates the corresponding 20 s time-domain SCG signals obtained from the estimated directions. The Modified-Capon method estimated the heart direction at 24°, which again produced the highest quality SCG signal. In contrast, MUSIC and Bartlett estimated the heart direction at −10°, and Capon estimated it at −3°. As shown in Figure 11, SCG signals from −10° and −3° directions exhibited inferior quality compared to those from 24°. These two experiments were conducted with the radar placed at different locations above the body, resulting in the heart appearing at varying angles relative to the radar antenna array. In both scenarios, the proposed Modified-Capon algorithm consistently identified the optimal heart direction and yielded SCG signals with the highest SNR. In contrast, the classical MUSIC, Capon, and Bartlett methods failed to accurately localize the heart. Notably, in the second experiment with the radar positioned above the head, all three classical methods estimated directions toward the head region instead of the heart, leading to poor SCG signal quality. These findings indicate that although classical beamforming methods perform well for large rigid targets, they are not well-suited for vital sign monitoring applications. The proposed Modified-Capon algorithm, tailored specifically for non-contact cardiopulmonary sensing, demonstrates superior performance in accurately estimating the heart direction and extracting high-SNR SCG signals.

#### 4.2.2 HRV analysis results

**FIGURE 12:**
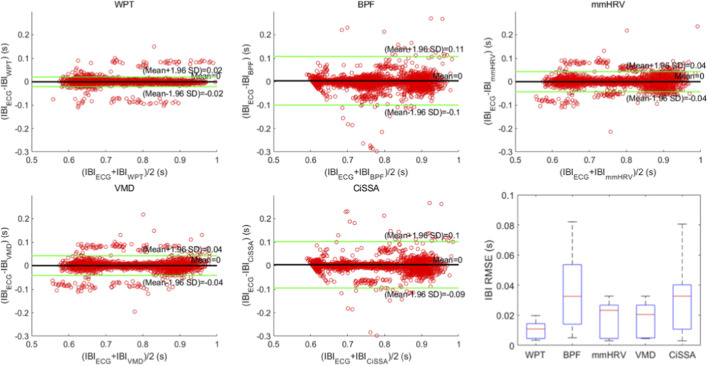
Bland-Altman plots and box plots of IBI sequences obtained by five heartbeat signal extraction methods.

Time-domain HRV analysis relies on calculating the IBI sequence on a beat-by-beat basis. Accurate IBI extraction is essential for precise calculation of HRV time-domain metrics. In this study, we applied the proposed heartbeat signal extraction method based on WPT and the AO point detection method to the collected data, calculating the IBI sequence and HRV time-domain metrics. To demonstrate the superiority of the proposed method, we conducted four comparative experiments, applying four widely used heartbeat signal extraction methods from recent radar-based vital sign measurement studies to the collected data. We then calculated the IBI sequence and HRV time-domain metrics using each method. These heartbeat signal extraction methods included the BPF method, mmHRV method, VMD method, and CiSSA method. All methods were evaluated against the ECG signal collected simultaneously as the ground truth reference. The ECG signal used the classic Pantokin algorithm (Pan and Tompkins, 1985) to detect R-peaks, experimental results are presented in Figure 12 and Table 2. Figure 12 displays Bland-Altman plots and boxplots of the IBI sequences obtained by the five heartbeat extraction methods. Table 2 presents the which were then used to calculate the IBI sequence and HRV time-domain metrics. The comparative true HRV time-domain metrics calculated from the synchronized ECG signal and the HRV time-domain metrics calculated from the IBI sequences obtained using each of the five heartbeat signal extraction methods. Results in Figure 12 indicate that the proposed method, which combines WPT-based SCG signal extraction with AO point detection, exhibits the best agreement with the reference IBI sequence from the ECG signal. The proposed method achieves the smallest 95% limits of agreement (LoA) interval, with an upper and lower LoA distance of 0.04 s. The mmHRV and VMD methods show slightly lower agreement with the ECG signal, each with a 95% LoA interval of 0.08 s. The CiSSA and BPF methods demonstrate somewhat lower consistency, with 95% LoA intervals of 0.19 s and 0.21 s, respectively. The final box plot in Figure 12 also demonstrates that the IBI sequence measured by the proposed method has the lowest root mean square error (RMSE) with the IBI sequence of ECG, along with the smallest interquartile range. This result indicates that the RMSE values of the IBI sequences measured by the proposed method compared to the ECG IBI sequence show minimal variation across different participants, reflecting a more stable IBI measurement. Table 2 presents the results of the three time-domain HRV indices (SDNN, RMSSD, and pNN50) calculated for 13 participants using five different heartbeat signal extraction methods. Additionally, Table 2 provides the absolute error values for each HRV index calculated by each extraction method compared to those obtained from the reference ECG method exhibits the smallest absolute errors for all HRV metrics among the five extraction methods. The average SDNN error for this method is 4.11 ms, the average RMSSD error is 8.05 ms, and the average pNN50 error is 2.15%. In comparison, the corresponding average SDNN errors for the mmHRV, VMD, and CiSSA methods are 16.16 ms, 16.26 ms, and 22.27 ms respectively. The average RMSSD errors for these methods are 19.82 ms, 17.32 ms, and 21.95 ms, while the average pNN50 errors are 10.95%, 11.1%, and 11.18%. The BPF method resulted in the highest absolute errors for the HRV metrics, with an average SDNN error of 27.57 ms, an average RMSSD error of 26.28 ms, and an average pNN50 error of 13.41%.

**TABLE 2:** HRV evaluation results of SDNN, RMSSD, and pNN50 of 13 subjects.

Metrics	Methods		User ID													Mean
			1	2	3	4	5	6	7	8	9	10	11	12	13	
SDNN	Value (ms)	ECG	43.75	30.02	32.02	31.45	26.05	27.14	31.03	26.08	29.15	28.28	20.44	17.24	46.74	∼
		**WPT**	54.59	38.35	29.40	35.07	33.56	28.83	31.48	28.50	30.16	29.82	24.79	21.31	51.68	∼
		BPF	64.74	51.29	29.38	39.56	36.58	35.73	133.45	52.31	25.37	108.00	72.52	27.46	58.61	∼
		mmHRV	61.60	56.34	46.29	37.83	33.70	29.73	33.48	29.53	28.00	31.87	76.11	31.68	101.05	∼
		VMD	63.56	59.17	29.38	39.78	35.98	41.04	33.64	30.18	30.26	31.89	73.79	28.99	97.87	∼
		CiSSA	65.85	51.72	29.71	44.19	34.22	48.61	126.88	53.39	25.32	29.48	70.51	27.76	58.98	∼
	Error (ms)	**WPT**	**10.84**	**8.33**	**2.62**	**3.63**	**7.51**	**1.69**	**0.45**	**2.42**	**1.01**	**1.54**	**4.35**	**4.07**	**4.94**	**4.11**
		BPF	20.99	21.27	2.64	8.11	10.53	8.59	102.41	26.23	3.78	79.72	52.08	10.22	11.88	27.57
		mmHRV	17.84	26.33	14.27	6.39	7.65	2.59	2.45	3.45	1.15	3.58	55.67	14.44	54.32	16.16
		VMD	19.80	29.15	2.64	8.33	9.93	13.90	2.60	4.10	1.10	3.61	53.35	11.75	51.13	16.26
		CiSSA	22.10	21.70	2.31	12.74	8.17	21.47	95.85	27.31	3.83	1.20	50.06	10.52	12.24	22.27
RMSSD	Value (ms)	ECG	19.53	23.21	13.82	14.42	16.20	13.86	19.95	15.46	14.62	20.91	5.65	5.52	53.06	∼
		**WPT**	39.55	24.84	13.82	26.29	28.61	23.31	22.05	22.35	17.89	23.79	15.31	17.04	65.95	∼
		BPF	52.65	43.55	14.00	38.52	36.91	28.86	96.00	40.59	15.35	93.48	32.29	20.71	64.90	∼
		mmHRV	49.64	46.38	47.89	37.11	34.45	21.11	23.87	25.40	23.81	30.11	32.08	30.82	91.26	∼
		VMD	51.51	47.95	13.87	41.26	36.12	29.52	24.17	26.93	21.31	30.03	33.62	24.31	80.71	∼
		CiSSA	51.97	43.78	15.44	42.41	35.94	40.05	92.97	42.85	15.25	24.89	29.53	21.39	65.05	∼
	Error (ms)	**WPT**	**20.01**	**1.64**	**0.48**	**11.87**	**12.41**	**9.45**	**2.10**	**6.89**	**3.27**	**2.88**	**9.66**	**11.53**	**12.89**	**8.08**
		BPF	33.12	20.35	0.19	24.10	20.70	15.01	76.05	25.13	0.73	72.57	26.64	15.19	11.84	26.28
		mmHRV	30.11	23.17	34.07	22.69	18.24	7.26	3.92	9.94	9.19	9.20	26.43	25.30	38.20	19.82
		VMD	31.97	24.75	0.06	26.84	19.92	15.66	4.22	11.47	6.69	9.12	27.97	18.79	27.65	17.32
		CiSSA	32.44	20.57	1.63	27.99	19.73	26.19	73.02	27.39	0.63	3.99	23.87	15.87	11.99	21.95
pNN50	Value (%)	ECG	0.59	1.73	0.23	0.13	0.00	0.46	1.51	0.11	0.36	0.57	0.00	0.00	0.42	∼
		**WPT**	6.69	3.48	0.13	4.22	5.87	2.30	1.93	2.04	0.65	1.55	2.40	2.17	0.47	∼
		BPF	12.89	8.18	0.27	14.36	9.69	2.55	29.41	13.21	0.17	42.98	1.67	1.03	43.72	∼
		mmHRV	11.87	12.26	25.81	13.18	8.02	1.27	1.89	3.73	2.13	4.51	1.56	7.49	54.72	∼
		VMD	12.44	10.50	0.13	16.60	9.09	32.96	2.10	4.75	3.43	4.41	1.56	2.30	48.75	∼
		CiSSA	13.31	10.21	0.81	16.49	9.89	4.43	31.46	14.63	0.21	1.09	1.48	1.26	45.90	∼
	Error (%)	**WPT**	**6.09**	**1.75**	**0.10**	**4.08**	**5.87**	**1.84**	**0.42**	**1.92**	**0.29**	**0.98**	**2.40**	**2.17**	**0.05**	**2.15**
		BPF	12.30	6.46	0.04	14.23	9.69	2.09	27.90	13.09	0.20	42.41	1.67	1.03	43.30	13.41
		mmHRV	11.28	10.54	25.57	13.04	8.02	0.81	0.38	3.62	1.77	3.94	1.56	7.49	54.30	10.95
		VMD	11.85	8.77	0.10	16.47	9.09	32.50	0.59	4.64	3.06	3.84	1.56	2.30	48.34	11.01
		CiSSA	12.72	8.48	0.57	16.36	9.89	3.98	29.95	14.52	0.15	0.52	1.48	1.26	45.48	11.18
Duration of data (min)			**10**	**10**	**10**	**10**	**10**	**10**	**10**	**10**	**10**	**10**	**10**	**10**	**10**	

Bold values indicate the best (lowest) absolute error among the compared methods.

We further reviewed recent related studies and summarized their results in Table 3. As shown in the Table 3, our method continues to demonstrate strong performance in terms of mean absolute error (MAE) and mean relative error (MRE) of the IBI sequence, as well as the errors in time-domain HRV metrics, including SDNN and RMSSD.

**TABLE 3:** Comparison of HRV analysis metrics of published papers and HRV analysis metrics of the method proposed in this paper.

References	Year	Radar type	Method	HRV analysis (error)
Wang et al. (2021b)	2021	77GHz FMCW	Heartbeat signal extractor for optimally decomposing the phase of channel information modulated by chest motion	$MAE\left(IBI\right)$ = 28 ms, ${SDNN}_{average}$ = 6.45 ms ${RMSSD}_{average}$ = 6.43 ms
Zhang et al. (2023)	2023	77GHz FMCW	Template Matching	$Median\;AE\left(IBI\right)$ = 12 ms
Chen et al. (2024)	2024	77GHz FMCW	Deep Learning	$MAE\left(IBI\right)$ = 14 ms
Sameera et al. (2024)	2024	2.4GHz CW	Wavelet-based signal processing enhanced with template matching	${SDNN}_{average}$ = 10.71 ms, ${RMSSD}_{average}$ = 14.16 ms
Dong et al. (2024)	2024	24GHz CW	Vectors analytic demodulation (VAD) method	${NRMSE}_{average}\left(IBI\right)$ = 1.196%
Shih et al. (2025)	2024	2.4GHz PQSIL	1. Respiration removal and autocorrelation 2. One-cycle segmentation 3. PRT shaping	${SDNN}_{average}$ = 4.91 ms, ${RMSSD}_{average}$ = 1.84 ms ${MRE}_{average}\left(IBI\right)$ = 1.97%
Vignoli et al. (2024)	2024	77GHz Cascade mm Wave radar	1. Beamforming to acquire multiple measurements from different points on the body 2. Physiology-inspired filter	${SDNN}_{average}$ = 2.48 ms ${RMSSD}_{average}$ = 3.45 ms
This work	2025	77GHz FMCW	Modified Capon beamforming 2. Wavelet packet transform 3. AO detection based on neighborhood search for envelope peaks	${MAE}_{average}\left(IBI\right)$ = 5.0 ms, ${MRE}_{average}\left(IBI\right)$ = 0.69% ${SDNN}_{average}$ = 4.11 ms, ${RMSSD}_{average}$ = 8.08 ms

* Median AE represents median absolute error. MAE represents mean absolute error. NRMSE represents normalised root mean square error. PQSIL is an abbreviation for phase and quadrature self-injection-locked.

## 5 Discussion and future work

This paper presents a non-contact HRV analysis method using a 77 GHz mm-wave radar. The proposed approach employs an optimized Capon beamforming algorithm to accurately localize the heart position, and combines WPT techniques to achieve high SNR non-contact SCG measurements. In addition, a precise AO point detection algorithm is developed. The integration of these techniques enables high-accuracy HRV analysis. SCG signals contain rich information related to cardiac mechanical activity and show great potential for future applications in cardiac function assessment and the diagnosis of arrhythmias and other heart diseases. This work thus contributes to advancing non-contact cardiac health monitoring and holds promising clinical application prospects. This study focuses on localizing the heart position of a single supine subject using beamforming techniques, followed by SCG signal extraction via wavelet packet transform, AO point detection, and HRV analysis. Scenarios involving multiple subjects are not considered. In the proposed heart localization method based on the optimized Capon beamforming algorithm, the beam scanning direction is limited to the central axis of the body, which constrains its applicability to various sleep postures. Employing 4D millimeter-wave radar with multidirectional beam scanning could potentially enable more accurate heart localization and yield SCG signals with higher quality. which will be considered as a future direction of this research. Moreover, the selected SCG template must have a sufficiently high SNR; otherwise, the localization performance may degrade, as the quality of all evaluated SCG signals is determined based on their DTW distance to the template.

## 6 Conclusion

This study proposes a high-precision HRV analysis method using FMCW radar. An optimized Capon beamforming algorithm is employed to accurately localize the heart position, effectively enhancing echo signals from the cardiac direction while suppressing clutter and interference from other directions. This provides a solid foundation for extracting high SNR’s SCG signals. By incorporating WPT, the method successfully separates the weak SCG signal from the stronger respiratory components. Additionally, a highly efficient and accurate AO point detection algorithm is developed to extract precise IBI sequences, further improving the accuracy of HRV analysis. We collected data from 13 subjects, each person collected data for 10 min. Experiments were conducted on this dataset and compared against four existing heartbeat signal extraction methods. The results demonstrate that the proposed method achieves superior performance in terms of both HRV analysis accuracy and computational efficiency.

## Data Availability

The raw data supporting the conclusions of this article will be made available by the authors, without undue reservation.

## Glossary

AO: Aortic opening
AoA: angle of arrival
ADC: analog-to-digital converter
BPF: Butterworth bandpass filter
CW: Continuous Wave
CiSSA: cyclostationary singular spectrum analysis
DOA: direction-of-arrival
DTW: dynamic time warping
DPD: decoding peak detection
ECG: electrocardiogram
EMD: empirical mode decomposition
EEMD: ensemble empirical mode decomposition
FFT: fast-time Fourier transform
FEnv: frequency envelope
FMCW: frequency-modulated continuous wave
HRV: Heart rate variability
HSMM: hidden semi-Markov model
IBI: inter-beat interval
IF: intermediate frequency
LoA: limits of agreement
MAE: mean absolute error
MRE: mean relative error
MIMO: multiple-input multiple-output
MDACM: modified differentiate and cross-multiply
MVDR: minimum variance distortionless response
MUSIC: multiple signal classification
ms: milliseconds
mmHRV: millimeter-wave radar HRV
PPG: photoplethysmography
PCG: phonocardiographic
pNN50: percentage of successive normal-to-normal intervals differing by more than 50 ms
rSCG: remote SCG
rPPG: remote PPG
RMSSD: root mean square of successive differences
SCG: seismocardiogram
SDNN: normal-to-normal intervals
SNR: signal-to-noise ratio
SQI: signal quality index
TDM: time-division multiplexing
UWB: Ultra-Wideband
VMD: variational mode decomposition
WPT: wavelet packet transform
2T4R: 2-transmit 4-receive
